# Mpox (Monkeypox) in Pregnant Women, the Placenta and Fetus: Correlation with Maternal-Fetal Transmission, Pathology and Strain Differences from MPXV Clades Ia, Ib, IIa, and IIb

**DOI:** 10.3390/v18040453

**Published:** 2026-04-09

**Authors:** David A. Schwartz

**Affiliations:** Perinatal Pathology Consulting, 490 Dogwood Valley Drive, Atlanta, GA 30342, USA; davidalanschwartz@gmail.com

**Keywords:** mpox, MPXV, monkeypox, mpox virus, congenital infection, Democratic Republic of the Congo, placental infection, stillbirth, emerging virus, orthopoxvirus, outbreak, mpox Clade I, mpox Clade II

## Abstract

Since the elimination of smallpox, mpox (monkeypox) is the most medically significant orthopoxvirus infection. As a result of numerous regional, national and global outbreaks of MPXV (mpox virus), there is an abundance of new data available on the effects of the different viral clades on clinical obstetrical and perinatal outcomes when infection occurs in pregnancy. In addition, there have been additional placentas from cases of congenital MPXV infection available for study. These recent data indicate that there are prominent differences between viral strains and their effects on the fetus, with MPXV Clade I strains (Ia, Ib) having the greatest risk for an adverse outcome in pregnancy, and Clade II strains (IIa, IIb) having far less risk. In particular, the ongoing outbreak of MPXV Clade Ib in the DRC indicates that there is a significant risk for adverse perinatal outcomes associated with infection in pregnancy, especially during the first trimester. These outcomes include spontaneous abortion, stillbirth, neonatal death and congenital mpox. The placenta in cases of congenital infection demonstrates abundant virus in the chorionic villi, with prominent involvement of Hofbauer cells. Similar to smallpox, transplacental transmission and adverse pregnancy outcomes are an important feature of certain strains of this orthopoxvirus infection when occurring in pregnant women.

## 1. Introduction

Mpox (formerly monkeypox) is an emerging zoonotic viral infection caused by the mpox virus (MPXV), which, since the eradication of smallpox, has become the most important member of the Orthopoxvirus genus from the standpoint of safety and public health [1,2,3]. For many decades, mpox had remained concentrated in equatorial Africa, where it circulated in 2 historically designated strains—the Congo Basin strain (later Clade I) and the West Africa strain (later Clade II). The Congo Basin strain was associated with human-to-human transmission, frequent viremia, and was endemic in the Democratic Republic of the Congo (DRC), where it caused sporadic outbreaks. It also occurred in Gabon, Sudan, the Republic of Congo, Cameroon, and the Central African Republic, and had a significantly higher case fatality rate than the West African strain. The West Africa strain had no human-to-human transmission, was much less prevalent, and occurred in Nigeria, Liberia, Sierra Leone, the Ivory Coast and other West African countries [4,5]. This nomenclature system existed until 2022, when geographic names were removed, and the former strains were designated as clades—Clade I and Clade II. In addition, MPXV Clade II was further subdivided into two genetically distinct strains, IIa and IIb, with Clade IIb identified as the etiological agent responsible for the 2022 MPXV global outbreak [6,7]. This outbreak occurred in non-endemic countries, the first time that the MPXV had traveled extensively outside of the endemic regions, and prompted the World Health Organization (WHO) to issue a public health emergency of international concern (PHEIC) in July 2022 [8]. The 2022–2023 mpox outbreak resulted in a global caseload of 92,781 confirmed cases in 188 countries [9], with the large majority transmitted via sexual contact by men-who-have-sex-with-men (MSM) and commonly presenting with anal/perineal genital lesions [10,11,12]. This outbreak also confirmed the pandemic potential of mpox.

In 2023, there were reports of increased numbers of new cases of mpox in DRC, including infections transmitted for the first time by sexual contact in South Kivu and Kwango provinces [13,14]. A novel strain of MPXV, termed Clade Ib, emerged in the Kamituga Health Zone of South Kivu province in September 2023 [15]. It was identified following genomic analysis of MPXV strains from previously non-endemic provinces in the eastern region of the DRC [15,16], and is suspected of being more transmissible than other clades [17]. The spread and intensity of MPXV infection in DRC and other African countries led to the WHO declaring a 2nd mpox PHEIC in August 2024 [18], with greater than 71,000 cases of suspected mpox occurring in DRC between January 2024 and February 2025 [19]. The circulation of all clades of MPXV in DRC through sexual networks has extended into 2026, with 367 confirmed cases in both men and women reported between 5 January and 15 February 2026 [20].

For many years, it had been known that the MPXV could result in adverse perinatal outcomes when occurring in pregnant women, but there were few cases available for analysis [4]. However, the ongoing mpox outbreaks in the DRC have resulted in numerous women of reproductive age becoming infected, which has provided an opportunity to investigate the effects of mpox on the mother, placenta, fetus and newborn. This communication examines the current knowledge of the effects of mpox in pregnant women in terms of placental, fetal and neonatal transmission, their association with MPXV clades and knowledge of the placental and fetal pathology in cases of vertical transmission, with a special focus on the pregnancy risks for the current circulating Clade Ib strain in the DRC.

## 2. Initial Cases of Mpox in Pregnancy

The first probable case of perinatal mpox infection was reported by Ježek and Fenner in DRC in 1988 [21,22,23]. Despite a lack of laboratory confirmation of MPXV infection, based on the clinical features, it was highly probable that it was a case of vertical transmission. The mother had clinical evidence of mpox at approximately 24 weeks’ gestation, with a premature infant delivered 6 weeks later with a generalized skin rash suggesting mpox.

The initial cases of confirmed mpox occurring in pregnant women were identified in the DRC during the Kole Human Monkeypox Infection Study that was conducted in the Sankuru Province between March 2007 and July 2011 [24,25,26]. Mbala and colleagues described four pregnant women with mpox who were treated at the General Hospital of Kole. MPXV infection was confirmed by polymerase chain reaction (PCR) in all cases. One mother had a moderate MPXV infection characterized by 76 skin lesions during the first trimester (6 weeks of gestation), was febrile and had a miscarriage 24 days after disease onset. Another pregnant mother developed a fever at 6 to 7 weeks of gestation, had severe mpox characterized by 1335 skin lesions, and had a miscarriage 2 weeks after the onset of fever. In the third case of mpox occurring during pregnancy, the mother had a fever at 14 weeks of gestation associated with mild infection (16 skin lesions) and delivered a full-term, uninfected infant that survived. The fourth mother had a stillborn infant with congenital mpox syndrome and is described below. Two of these women had first-trimester miscarriages, which are relatively common in the general population. However, given the fact that both mothers had moderate to severe mpox during their pregnancies, and based upon our current knowledge of the potential for MPXV to result in transplacental infection, it appears likely that both fetal losses were due to this disease.

In addition to these four cases in the DRC, pregnant women having mpox were also described from Nigeria. Two pregnant women having mpox were reported during the 2017–2018 mpox outbreak in Nigeria [27,28]. A pregnant woman having clinical symptoms of mpox had a spontaneous abortion at 26 weeks of gestation, but no laboratory testing was performed, and the fetus was not described [27]. In the second case, a pregnant woman with mpox at 16 weeks of gestation had preterm rupture of membranes and a spontaneous abortion, also with no laboratory testing [28].

## 3. First Case of Congenital Mpox Syndrome

**Figure 1:**
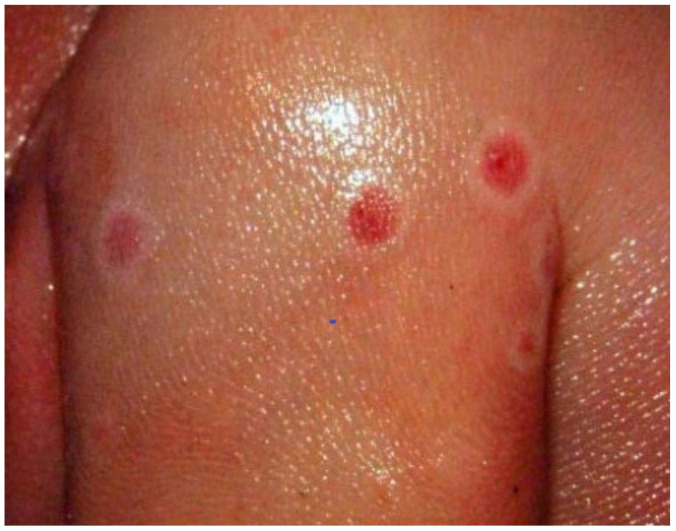
Mpox lesions on the skin of a 28-week stillborn infant with PCR-confirmed MPXV infection. Photograph courtesy of Phillip R. Pittman, MD.

A pregnant woman enrolled in the Kole Human Monkeypox Infection Study developed a new onset of fever at 18 weeks of gestation, became symptomatic with a moderate mpox infection and fever, and had 133 skin lesions [24,26]. A stillborn infant was delivered 21 days following the onset of fever. Various maternal and fetal specimens, including umbilical vein blood, fetal skin lesions, sterile peritoneal fluid, and placenta, were positive for MPXV by PCR. Although genomic analysis was not performed, it is almost certain that this was MPXV Clade I (now Clade Ia) that was endemic in the region. An autopsy was performed, which demonstrated nonimmune hydrops fetalis and diffuse pale pink to white maculopapular lesions (pox). These lesions were present on the face (scalp, forehead, nostrils and cheek), skin of the extremities, soles of the feet, palms of the hands, chest, shoulders, buttocks and abdomen. Many lesions were bright red and surrounded by white halos, and some had superficial ulcerations (Figure 1).

**Figure 2:**
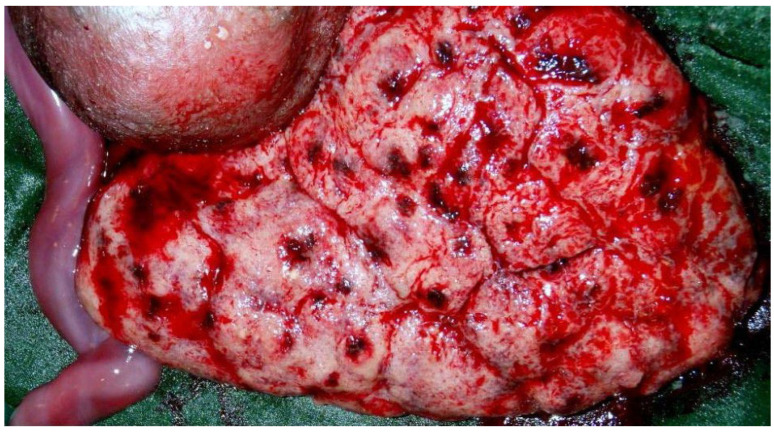
Punctate hemorrhages of the maternal (basal) surface of a placenta from a stillborn infant following congenital transplacental MPXV transmission in the DRC. Photograph courtesy of Phillip R. Pittman, MD.

**Figure 3:**
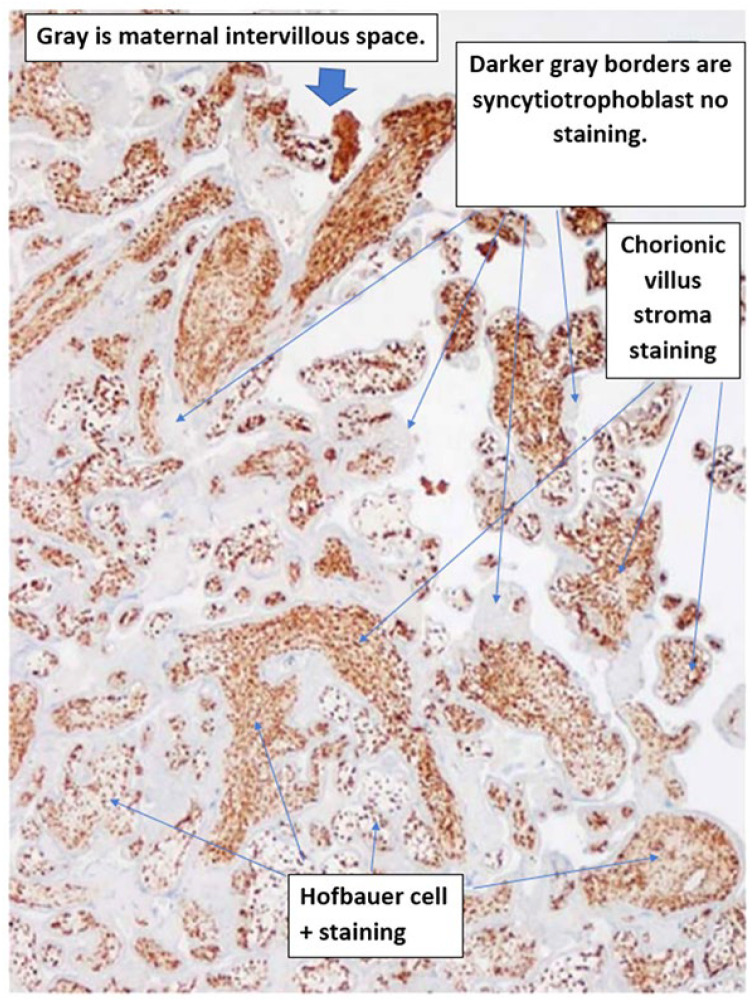
Placenta of a stillborn infant with confirmed congenital mpox infection. Strong diffuse positive staining (brown) for orthopoxvirus antigen in Hofbauer cells in the chorionic villi. Immunohistochemistry with an antibody to vaccinia virus. Original magnification ×10. Photograph courtesy of Phillip R. Pittman, MD.

No pox lesions were present in the internal organs, but examination of the abdomen revealed ascites and hepatomegaly. The placenta had abnormal punctate hemorrhages on the maternal (basal) surface (Figure 2). Immunohistochemistry of select tissue sections was performed using a polyclonal antibody to another orthopoxvirus, vaccinia virus (VACV), which exhibits cross-reactivity with MPXV. There was strong positive staining for anti-VACV antibody in sections of the skin, liver and placenta. The placenta showed intense positive staining diffusely in the chorionic villi within stromal cells. These cells, termed Hofbauer cells (Figure 3), were villous macrophages of fetal origin and were increased in number, a pathological process termed Hofbauer cell hyperplasia.

## 4. Pregnancy and Maternal-Fetal Transmission of MPXV Clade IIb During the Global 2022–2023 Outbreak

The global MPXV outbreak of 2022–2023 affected 115 countries, areas, and territories and resulted in 92,783 cases and 171 deaths as of 30 November 2023 [29]. Compared with previous mpox outbreaks, the 2022–2023 outbreak was unique in that it was the first time mpox had spread widely outside of the endemic countries in West and Central Africa; it was transmitted mostly by sexual contact; and most persons affected were gay and bisexual men (men who have sex with men, MSM). Genomic analysis found that the MPXV strain responsible for the majority of the cases was a variant of the Clade II virus but possessing a sufficient number of new mutations to be categorized as a new clade, termed Clade IIb (now termed Clade IIb lineage B.1) [30,31,32].

Although 94% of cases occurred in MSM, some women and children were affected. The WHO reported mpox occurred in 58 females who were pregnant or recently pregnant as of 13 June 2023 [33]. Their median age was 28 years, with MPXV infection occurring in all 3 trimesters—5 cases in the 1st trimester, 12 cases in the 2nd, and 10 cases in the 3rd trimester; gestational age data were unavailable for 31 individuals. Mpox occurred in an individual who was 6 weeks or less postpartum. There were 13 women who required hospitalization, but none needed intensive care, and no maternal deaths occurred. In the nine cases where the manner of mpox transmission was known, the most frequent exposure was a sexual encounter (four cases). In cases where the risk factor for maternal infection was known, sexual transmission of MPXV was the most common. There were 2 neonates—one with confirmed mpox and one probable—identified as having congenital infection.

In Brazil, there were 22 pregnant women with confirmed or probable mpox during the period from June 2022 to 30 April 2023: 2 first-trimester infections, 11 second-trimester infections and 8 third-trimester infections [33,34]. One pregnant mother was hospitalized for clinical management, and one was hospitalized for isolation. In the United Kingdom, a neonate with mpox resulting from peripartum transmission within a family cluster was also reported, in which transplacental transmission could not be excluded [35]. The United States reported the largest number of pregnant women having mpox during this outbreak—21 cases, all of whom had a rash. There were three cases that occurred during the first trimester, four cases during the second, three during the third trimester, and two cases postpartum that developed within 3 weeks of pregnancy. Female genital lesions were observed in four women, but none near the time of delivery. Although four women had symptoms that required hospitalization, none had an unplanned delivery or needed intubation or intensive care. Among the three cases having known infant outcomes, there was one spontaneous abortion at 11 weeks of gestation, and two full-term births with no viral transmission or complications. Three mothers had mpox symptoms within 3–4 days following delivery, and their infants developed lesions within one week of the onset of their symptoms [36].

## 5. MPXV Infection in Pregnant Women, Fetuses and Neonates in the Current Clade Ib Outbreak in DRC

As the outbreaks of MPXV Clade II waned, beginning in 2023, there was an alarming surge of MPXV Clade I cases in the DRC with a record high of 14,626 new cases and 654 fatalities in that year [37]. A new strain, termed the MPXV Clade Ib, emerged in the South Kivu province of DRC, demonstrating sustained human-to-human transmission, including sexual contact, and spreading eastward to Uganda, Burundi, Rwanda, Kenya, and Zambia [38]. During the period September 2023 to February 2024, the mining town of Kamituga in South Kivu province recorded more than 200 new cases of mpox and became the epicenter for the outbreak. The DRC Ministry of Public Health declared a mpox epidemic on 24 April 2024. In 2024, the MPXV Clade Ia strain was found to be co-circulating with Clade Ib in Kinshasa, the largest city and capital of DRC having over 17 million residents [32]. On 14 August 2024, the WHO declared a global health emergency due to the newly identified Clade Ib outbreak in eastern DRC [32]. The MPXV outbreak centered in the DRC has continued into 2026, and has been characterized by numerous cases of pregnant women becoming infected as well as by maternal-fetal transmission occurring with adverse perinatal outcomes.

Vakaniaki et al. reported 3 cases of maternal-fetal transmission of Clade Ib MPXV infection from the DRC [39]. In the first case, a pregnant 20- to 30-year-old multiparous woman became febrile at 6 weeks of gestation and developed a generalized rash, genital edema and pain, inguinal adenopathy, vaginal discharge, labial edema and generalized pruritis. She reported having previous sexual contact with a person suspected of having mpox. On admission to the hospital, she had 100 to 250 vesiculopustular skin lesions and tested positive for Clade Ib MPXV by PCR. At 8 weeks of gestation, she developed abdominal pain accompanied by vaginal bleeding and had a spontaneous abortion. MPXV nucleic acid was identified from both embryonic and placental tissues, as well as a maternal skin swab. Pathology evaluation using both immunohistochemistry and immunofluorescence revealed MPXV antigen in CD68-positive placental villous macrophages (Hofbauer cells) and in alpha-fetoprotein-positive embryonic cells, indicating transplacental transmission. In the second case, a 25- to 35-year-old pregnant woman at 16 weeks of gestation developed diffuse vesiculopustular lesions, fever, and dysphagia. She was HIV positive and receiving antiretroviral therapy. Two weeks prior to hospitalization, she had sexual contact with her husband, who had skin lesions consistent with mpox. She became febrile and had dysphagia one week prior to admission, followed by inguinal lymphadenopathy, the onset of skin lesions spreading from the genital area to other parts of her body, and genital pain. She developed over 250 skin lesions, consistent with mpox, which gradually subsided. Fetal movements ceased fifteen days after she tested positive for MPXV Clade Ib, and an ultrasound confirmed intrauterine fetal demise at 18 weeks of gestation. The stillborn fetus was delivered by cesarean section, at which time the uterine cavity was noted to have several dark, vesicular lesions. The fetus had mpox-like lesions on the face, chest, abdomen, and upper limbs. MPXV DNA was identified in placental and fetal tissues. In the third case, a 20-to-30-year-old HIV-positive pregnant woman developed fever, dysphagia, genital ulcers and generalized mpox skin lesion at 34 weeks of gestation. She reported having skin-to-skin contact with a family member having mpox two weeks prior to admission. She tested positive for MPXV Clade Ib and was admitted to the hospital for 29 days. Thirteen days after her discharge (40 weeks of gestation), she delivered a live-born infant with multiple ulcerative skin lesions. PCR testing for MPXV DNA was positive in swabs of the newborn’s oropharynx and placenta. Pathology immunohistochemical evaluation of the placenta demonstrated MPXV antigen present in CD68-positive cells of the chorionic villi, consistent with Hofbauer cells. The infant died at 3 months of life, probably from an unrelated cause. It was reported that the pathology examination of all three placentas did not show evidence of inflammatory abnormalities (such as villitis or intervillositis).

During the period from June to October 2024, Bugeme and colleagues investigated 973 suspected cases of MPXV Clade Ib in Uvira, a trading city on the northern shores of Lake Tanganyika in South Kivu Province [40]. Among the study patients, there were 131 females of reproductive age, with 19 (14.5%) self-reporting as being pregnant. Seven of the 19 pregnant women were in their third trimester, with three having severe to very severe suspected mpox. There was follow-up data for nine pregnant women—four tested positive for MPXV by PCR, one tested negative, and four had no laboratory results. The mothers delivered nine newborns (one set of twins), all liveborn, with no reported complications or skin lesions. There was a single first-trimester stillbirth reported in a woman testing positive for MPXV. There were also two additional women who were still pregnant at the time of follow-up, with no reported complications.

Providing further documentation of the significant association of MPXV Clade Ib infection during pregnancy with fetal loss in the DRC is the report of Vakaniaki et al. from 2026 [41]. This prospective cohort study analyzed pooled data from three cohort studies (Uvira mpox, PREGMPOX and MBOTE-SK) and a randomized controlled trial (PALM007). These studies were conducted in the Sankuru, Maniema and South Kivu provinces of DRC between 29 December 2022 and 20 June 2025. The authors enrolled 89 pregnant women and adolescent girls with a PCR-confirmed diagnosis of mpox, collected sociodemographic data, clinical medical and obstetrical history, laboratory results, and mpox exposure factors, and followed their hospitalizations, delivery and discharge into the post-partum period. The occupational history of the participants included housework in 35 (39%), agricultural activity in 24 (27%), commercial activity in 18 (20%), and commercial sex work in 4 (4%). Clinical outcome covariables were evaluated that included (1) spontaneous or missed abortion; (2) stillbirth; (3) preterm birth; (4) live birth with macroscopic mpox-like lesions; (5) early neonatal death (up to day of life 7), (6) congenital anomalies; and (7) maternal death. Among the 89 pregnant study patients, there were 25 in the first trimester (28%), 31 in the second trimester (35%), and 33 in the third trimester (37%). Pregnancy outcomes were available for 69 patients (78%). Poor perinatal outcomes were found in 35 (51%) women (95% CI 38–63), which included fetal loss in 31 patients (45%; 95% CI 33–57). These fetal losses were the result of spontaneous abortions in 16 (52%), missed abortions in 4 (13%), and 11 women (35%) having stillborn infants. There were 38 liveborn infants in this cohort study, with four having mpox-like lesions consistent with congenital intrauterine infection; one of these neonates died a few hours after delivery. There were no maternal deaths, preterm deliveries or congenital anomalies identified. The investigators found that the rates of adverse infant outcomes differed by gestational age, with first trimester maternal MPXV infection have a significantly greater risk of adverse pregnancy outcomes than did MPXV infection in the second trimester ([RR] 0.6 [95% CI 0.4–0.9]) and third trimester ([RR] 0·2 [95% CI 0.1–0.4]) trimesters (*p* = 0.0008). Adverse obstetrical outcomes were also found to be associated with direct sexual contact with the index case, high MPXV load in skin lesions, positive maternal HIV status, and the occurrence of maternal genital lesions.

## 6. Placental Pathology

The placenta is the largest fetal organ and is the most important barrier to transmission of infectious agents between an infected mother and her unborn infant. The first placenta to be evaluated from a case of congenital MPXV infection was the MPXV placental infection discussed previously in Section 3, occurring in a stillborn infant from the DRC having congenital mpox acquired from a mother with active infection. In this case, originally described by Mbala et al. [24], the placenta was fortunately saved and submitted for pathological evaluation. Schwartz and colleagues [4,22,26,42] subsequently described details of the features of this important specimen, although their ability to thoroughly investigate the pathology was limited. Immunohistochemical staining demonstrated an increased number of chorionic villous stromal macrophages, or Hofbauer cells, which were increased in number (Hofbauer cell hyperplasia). Strong positive intracytoplasmic staining in the Hofbauer cells for poxvirus antigen using an antibody to a cross-reactive orthopoxvirus, vaccinia virus, was identified (Figure 2). There was no evidence of villitis or other inflammatory pathological abnormalities. Presumably, this intrauterine congenital fetal infection was caused by what would now be termed MPXV Clade Ia. Based upon these observations, Schwartz et al. [43] proposed a mechanism for the transmission of MPXV during a period of maternal viremia in which MPXV travelled from the maternal circulation into the placenta via the uterine vessels, followed by transplacental transmission of the virus into the fetal chorionic circulation and the fetus. During this process, MPXV would utilize an as-yet unknown attachment site to fix to and penetrate the trophoblastic barrier of the chorionic villi, enter the chorionic villous stroma, where it would be exposed to Hofbauer cells, and eventually breach the fetal-derived villous capillary circulation to reach the fetus. This proposed mechanism of transplacental infection is not unique, and occurs together with involvement of Hofbauer cells with other viral infections, including SARS-CoV-2 [44], Zika virus [45], and others [46]. Thus far, examination of placentas from pregnant mothers having mpox with intrauterine MPXV transmission resulting in infants having congenital mpox have supported this hypothesis. In addition, preliminary evidence from the few placentas described from congenital MPXV infections suggests that the pathology of MPXV differs significantly from that of some other viruses. Unlike SARS-CoV-2 infection, which can result in extensive placental destruction, inflammatory changes and hemorrhage, MPXV does not appear to cause similar placental damage [47,48]. Placentas infected with MPXV have demonstrated high viral load using immunohistochemistry, which is unlike placentas from cases of congenital Zika virus transmission, in which viral load is low [45].

The recent report of Vakaniaki et al. [39] is of special significance in that it provided an additional three placentas for pathological analysis from MPXV-transmitting maternal-fetal dyads in the DRC. These represent the first placentas to be examined from congenital MPXV Clade Ib infection. All three placentas were found to be positive for MPXV antigen using immunohistochemistry, demonstrating positive viral staining in the Hofbauer cells and corroborating the previous findings of Schwartz and colleagues.

## 7. Discussion

Mpox continues to occur in DRC, where concurrent infections with multiple MPXV strains are present. In cases of MPXV Clades Ia and IIa, zoonotic transmission plays an important role. For MPXV, whereas Clades Ib and IIb, the spread of the virus is occurring through sustained human-to-human transmission without zoonotic exposure. In non-pregnant patients, the severity of the disease is greater for Clade Ia infections, with an average case fatality rate up to 12%, when compared with other MPXV clades that have case fatality rates between 0 and 3.6% [49]. In regions endemic for Clade Ia, transmission remains predominantly zoonotic, and in particular, results in significant morbidity and mortality in children less than 5 years [49]. Table 1 summarizes the major epidemiological characteristics of MPXV strains and current knowledge on their effects in pregnant women, the fetus and the placenta.

There are now numerous examples of transmission of MPXV from an infected mother to her fetus through the placenta, with viral load reaching up to 10^6^ copies per milliliter [50]. The hypothesis of Schwartz and colleagues that the mechanism for transplacental transmission of MPXV results in viral infection of the chorionic villi, and that Hofbauer cells are permissive to MPXV infection and are an important factor in maternal-fetal transmission, has now been documented from multiple placentas in cases of congenital infection [22,25,26,39]. The ongoing outbreak of MPXV Clade Ib in the DRC indicates that, similar to smallpox, transplacental transmission and adverse pregnancy outcomes seem to be an important feature of orthopoxvirus infection when occurring in pregnant women.

Although the number of fetuses and newborn infants with congenital mpox available for study has been limited, thus far MPXV has not demonstrated evidence of neurotropism toward fetal brain stem or neural progenitor cells, and no congenital brain abnormalities have been documented to date. Thus, it appears to be different from some other congenital viral infections that affect the central nervous system, such as cytomegalovirus, Zika virus, rubella and herpes simplex virus.

Perhaps the most important finding from all of these reports is confirmation of the differences in the potential lethality of specific clades of MPXV when infection develops in pregnant women [4]. The first study of mpox in pregnant women showed a high perinatal mortality rate of 75%, but was limited to only 4 cases from presumed MPXV Clade Ia infection [24]. In contrast, at least 58 cases of pregnant women infected with mpox occurred during the 2022–2023 global mpox outbreak [33], but no confirmed cases of fetal death occurred, and there was only a single laboratory-confirmed case of congenital infection. The current MPXV Clade Ib outbreak in the DRC is proving to be highly risky for pregnant mothers who acquire the infection. The investigation of pooled clinical outcomes data by Vakaniaki et al. [41] found that almost 51% of pregnant women infected with Clade Ib had an adverse outcome, and nearly one-half of the mothers sustained a fetal loss. When MPXV infection occurred in the first trimester, there was an adverse outcome in 94% of pregnancies. Four of 38 liveborn infants had congenitally acquired MPXV infection.

The initial cases of recombinant MPXV strains have recently been reported. In December 2025, the first reported case of a clade Ib/IIb MPXV recombinant strain was identified in Great Britain [51]. Just after this, in January 2026, the National IHR Focal Point (NFP) of India alerted the WHO of a traveler with mpox who had a recombinant viral strain with genomic elements of both clades Ib and IIb MPXV [52,53]. Both of these infections occurred in non-pregnant adults, and it remains to be seen what the features of a recombinant MPXV strain will have on pregnant women, the placenta and fetus.

These data indicate that mpox in pregnancy is an important public health problem that is likely to continue. The pandemic potential of MPXV strains and their capability to spread throughout the world, as evidenced in the 2022–2023 outbreak, indicate that this infection is not restricted to only endemic countries. There must be increased human and financial resources allocated to investigating and preventing MPXV in pregnant women. It is hoped that newly implemented MPXV vaccine strategies will prevent maternal-fetal MPXV transmission, similar to the success that vaccination for SARS-CoV-2 has had in preventing placental infection and stillbirth from COVID-19 [54].

## Figures and Tables

**Table 1:** Epidemiological features and pregnancy effects of the major MPXV clades.

MPXV Clade	Geographic Distribution	Virulence	Transmission Patterns	Pregnancy Effects
**Ia**	Central/Eastern Africa; major outbreaks in DRC & Madagascar (2025–2026)	Most virulent; Variable CFR from 1.4% to 10% based on the study; Higher CFR in children <15 years	Zoonotic and human- to-human; Includes community transmission in multiple African countries	Original 4 cases of maternal infection (Congo Basin strain) with 75% perinatal mortality; Placental infection; Transplacental transmission; Stillbirth; Miscarriage; Congenital infection
**Ib**	Central/Eastern Africa; also detected in travel-associated cases in the EU/US since 2024	Lower CFR (<0.5%) than Clade Ia; Virulent in pregnancy	Human-to-human; Some atypical presentations	Maternal-fetal transmission; Placental infection; Spontaneous abortion; Extensive fetal loss in the current DRC outbreak
**IIa**	West Africa (Ghana, Guinea, Liberia) and sporadic global cases	Lower virulence than Clade I	Zoonotic; Limited human-to-human transmission	Two cases of fetal loss in the 2017–2018 Nigeria mpox outbreak (West Africa strain) with no lab testing
**IIb**	Global outbreak lineage (2022–present); widespread in the US, Europe, Latin America and Asia	Lowest CFR (~0.2%) globally, but can be higher in immunocompromised groups (e.g., HIV)	Highly efficient human-to-human; Intimate sexual contact; Atypical anogenital clinical presentation common	Many infected pregnant women reported during the global 2022–2023 outbreak; Rare congenital infection; No maternal deaths

## Data Availability

No new data were created or analyzed in this study.
